# Supplementary material for: Proximal CA1 20–40 Hz power dynamics reflect trial-specific information processing supporting nonspatial sequence memory
Source: eLife. 2022 May 9;11:e55528. doi: 10.7554/eLife.55528 (PMC9170241; doi:10.7554/eLife.55528)
Supplement: Supplementary file 1. — Values in parentheses indicate trials included in the analyses (after artifact rejection; counts from most proximal electrode). [file elife-55528-supp1.docx]

**Supplementary File 1**

| Well-trained | | | | |
| --- | --- | --- | --- | --- |
| **Animal** | **InSeq+** | **OutSeq+** | **InSeq-** | **OutSeq-** |
| 1 | 135 (55) | 25 (24) | 5 (2) | 5 (2) |
| 2 | 123 (102) | 22 (20) | 12 (12) | 6 (6) |
| 3 | 105 (91) | 11 (11) | 6 (6) | 10 (9) |
| 4 | 135 (131) | 27 (27) | 14 (13) | 17 (14) |
| 5 | 164 (164) | 16 (16) | 25 (25) | 14 (14) |
| **Novel2** | | | | |
| **Animal** | **InSeq+** | **OutSeq+** | **InSeq-** | **OutSeq-** |
| 1 | 155 (82) | 19 (16) | 13 (12) | 26 (16) |
| 2 | 88 (83) | 9 (8) | 7 (7) | 22 (20) |
| 3 | 136 (86) | 3 (3) | 11 (10) | 15 (12) |
| 4 | 142 (141) | 2 (2) | 12 (11) | 26 (36) |
| **Novel1** | | | | |
| **Animal** | **InSeq+** | **OutSeq+** | **InSeq-** | **OutSeq-** |
| 1 | 75 (51) | 3 (2) | 7 (5) | 23 (12) |
| 2 | 58 (57) | 1 (0) | 5 (5) | 11 (11) |
| 3 | 92 (75) | 0 (0) | 8 (7) | 11 (9) |
| 4 | 74 (71) | 2 (2) | 5 (5) | 14 (12) |
